# Validation of Health Extension Workers Job Motivation Scale in Gamo-Gofa Zone, Southern Ethiopia: A Cross-Sectional Study

**DOI:** 10.1155/2015/250610

**Published:** 2015-02-24

**Authors:** Shikur Mohammed, Marelign Tilahun, Mesfin Kote, Mohamedaman Mama, Dessalegn Tamiru

**Affiliations:** ^1^Department of Public Health, Arba Minch University, P.O. Box 21, Arba Minch, Ethiopia; ^2^Department of Population and Family Health, Jimma University, Jimma, Ethiopia

## Abstract

*Introduction.* Well-trained and highly motivated community health workers are critical for delivery of community-based health care services. Understanding what motivates especially community health care providers for better community health requires the use of psychometrically reliable and valid scale. This study was conducted to validate job motivation scale in Gamo Gofa Zone, Southern Ethiopia. *Methods.* A cross-sectional study was conducted in 150 health care posts in Gamo Gofa Zone, from February 01, 2013, to March 01, 2013. A total of 301 participants responded to questionnaires asking about sociodemographic characteristics and job motivation. Exploratory factor analysis with principal component extraction and varimax with Kaiser Normalization rotation were employed to develop scales for job motivation. Eigenvalues greater than 1 were used as criterion of extraction. Items with item factor loadings less than 0.4 and double loaded items were dropped. Alpha and exploratory factor analyses were examined to test reliability and validity of the scale. *Results.* During exploratory factor analysis eight factors emerged from the three dimensions of job motivation scale, namely, educational career, workload, financial incentive, supervisor encouragement, community recognition, access to infrastructure, living condition, and better achievement in work. The factor loadings of the items in each dimension ranged from 0.58 to 0.83. Crobach's alpha of the scales ranged from 0.79 to 0.90. To check validities of the scales developed in this study, the previous studies conducted to develop job motivation scale were used. *Conclusion.* Although the present scale has the potential to measure the job motivation of health extension workers and it is low in cost and easy to administer and analyze, it should be field-tested at different settings.

## 1. Introduction

Recognizing the huge gap between the need and health care services available, Ethiopia launched new health extension program (HEP) to provide health care services in accessible and equitable manner to all segments of the population with special attention to mothers and children. Moreover, the majority of the health problems in Ethiopia are due to infectious diseases, which are better managed by an approach that focuses on preventive and promotive health services [1, 2].

As a preventive health program, the HEP promotes four areas of care: disease prevention and control, family health, hygiene and environmental sanitation, and health education and communication [1].

Health extension workers (HEWs) are the cadre for the implementation of health extension program. All HEWs are women at least 18 years of age with a minimum of 10th grade education recruited from the communities in which they will work, who are trained to implement health extension packages of 16 health care activities at the kebele (village) level and must complete a one-year course of training. Upon completion of training they are assigned in pairs in kebele where they were recruited first and work directly with individual families to serve 500 households. They are expected to spend 75 percent of their time visiting families in their homes and performing outreach activities in the community. The remaining 25 percent of their time is expected to be spent providing services at the health posts, like immunizations, family planning services, and others [3].

To address community demands for basic curative care, HEWs are also trained to provide first aid, treat malaria, dysentery, and intestinal parasites, and refer cases to the nearest health center when more advance care is needed [1, 3].

The health care provider motivation has often been identified as a central problem in health service delivery and quality [4, 5]. Low motivation leads to insufficient translation of knowledge, underutilization of available resources, and weak health system performance. Poor worker motivation can manifest as tiredness, absenteeism, poor process quality, turnover rate, high vacancy rates, and indifferent performance [5].

Study conducted in Ethiopia revealed that poor transportation and communication systems, inconsistent availability of minimum standard of equipment and furniture, salary level which seems inadequate, and not being appreciated by the colleagues demotivated health extension workers [6]. However, this has been conducted where the career structure, upgrading, promotion, and rewards for health extension workers had not been formally established.

The successful delivery of lifesaving programs depends on the motivation and effective job performance of health workers [4]. Therefore, this study was done to validate health extension workers job motivation scale in Gamo-Gofa Zone.

## 2. Methods

### 2.1. Study Settings

The study was conducted among health extension workers in Gamo-Gofa Zone, Southern Ethiopia. The total area of Gamo-Gofa Zone is about 12,004.26 sq/km. The zone has 17 districts (15 rural districts and 2 urban district administrations) and 482 kebeles (448 rural kebeles and 34 urban kebeles) with total population of 1,837,896 [7, 8].

The zone has 3 hospitals, 66 health centers, 471 community health posts, and 125 private clinics and the health service coverage at health center and health post level is 100%. There were 831 health extension workers in the zone where 763 were rural health extension workers [7].

### 2.2. Study Design and Population

A cross-sectional study design was used. All health extension workers who were working in Gamo-Gofa Zone were the source population; and all health extension workers who were working in selected districts of the zone were the study populations.

### 2.3. Sample Size and Sampling Procedure

The sample size was determined using single population proportion formula based on 31% proportion [9], marginal error of 5% (*d* = 0.05) and standard score corresponding to 95% confidence (*Z*_*α*/2_ = 1.96) and design effect of 1.5. By applying the correct formula the final sample size was found to be 295.

In Gamo-Gofa Zone there are 15 rural districts of which 5 (30%) rural districts were selected using cluster sampling technique. Since the sampling technique was cluster sampling technique, all HEWs within the selected districts were included in the study. On average there were 30 kebeles in each district and 2 health extension workers in each kebele. So the required sample size was attained from the selected districts. List of HEWs in the selected districts was obtained from the respective district health offices.

### 2.4. Instruments and Measures

The questionnaires were developed in English after reviewing different literatures. The questionnaire contained sociodemographic variables (age, year of service, marital status, religion, monthly income, educational status, ethnicity, and additional source of income) and items (achievement in work, satisfaction with the work done, working hard towards quality service, interest to provide community services, satisfaction on monthly salary, satisfaction on field allowance, satisfaction on housing benefits, supervisor encouragement in decision making and working hard, supportive feedback, opportunity for upgrade/trainings, altruism, self-efficacy, community perception, relationship with local community, families' area of residence, access to transportation, water sources and market place, and convenient roads to move in the community) to measure work motivation [10, 11].

Amharic translated self-administered questionnaires were used to collect the data. Each question explored their level of motivation on a Likert scale of 1 (strongly disagree) to 5 (strongly agree). The construct of the questions was balanced with both positive and negative directions to prevent similar responses. The questionnaires were then pretested on the health extension workers in the health posts found in Arba Minch town (not included in the study), making up 5% of the study population, before the actual data collection began. Finally the Amharic questionnaires were translated back into English to ensure semantic equivalence.

Factor analysis with principal component extraction and varimax Kaiser Normalization rotation methods were employed to develop the job motivation scale. To determine factors “*eigenvalue*” was used and factors with values greater than 1.0 were kept and the rest discarded. Accordingly, eight factors emerged. After the appropriate number of factors had been determined, a factor correlation matrix was done to identify the relationship between the items and the factors. A value of 0.40 was taken as the lower cut-off point for factor loading. When naming the factors, items that have higher factor loadings were taken as a base. The reliability of factors was checked against Cronbach's alpha greater than 0.7.

### 2.5. Data Processing and Analysis

Data were checked for completeness and were entered into Epi-info and then the data were exported to SPSS software (version 16.0; SPSS Inc., Chicago, IL) for analysis. The data were explored using descriptive analyses to clean data-entry errors. Factor analysis was then conducted to create scales for job motivation. Assumptions of factor analysis, including sampling adequacy and multicollinearity, were checked. Alpha and exploratory factor analyses were examined to test reliability and content validity of the scale.

### 2.6. Ethical Consideration

The research proposal was approved by the ethical review committees of Arba Minch University, College of Medicine and Health Sciences. Permission letter was obtained from the Gamo-Gofa Zone health department and the respective district health offices. Oral informed consent was obtained from the study participants before the commencement of data collection. The right of study participants to refuse participation or withdraw from the study at any point was respected. All accessed data were kept confidential and only accessed by the study teams. Data were not shared with anybody in a way respondents' individual identity was identified.

## 3. Results

### 3.1. Characteristics of Respondents

A total of 301 HEWs were included in this study although the proposed sample size was 295. In this study complete response was obtained from all (100%) respondents. The mean age of the respondents was 26.2 (SD ± 3.3). The majority of study participants 196 (65.1%) were married and 216 (71.8%) attended 10th grade. About 30% of the study participants did not have any other personal sources of earning. The mean service year of study participants was 5.1 (SD ± 2.1) (Table 1).

### 3.2. Scale Development

Job motivation contained three dimensions: institutional dimension, societal dimension, and individual work performance dimension. During exploratory factor analysis, four factors emerged from the institutional dimension. In this dimension, educational career, workload, financial incentives, and supervisor encouragement explained 26.3%, 11.4%, 18.7%, and 24.6% of the total variance, respectively. The numbers of the items were four for educational career, two for workload, four for financial incentives, and four for the supervisor encouragement. In general, factor loadings of the institutional dimensions items on their respective factors ranged from 0.61 to 0.83 (Table 2). Cronbach's alphas of the four scales were 0.91, 0.88, 0.85, and 0.80, respectively (data not presented).

There were three factors that emerged from the societal dimension, which accounted for 33.5% (community recognition), 31.3% (infrastructures accesses), and 22.3% (living condition) of the total variance, respectively. Community recognition had three items. Infrastructures access had five items. Living condition had four items. Generally, the factor loadings of the societal dimension items ranged from 0.66 to 0.77 (Table 3). Cronbach's alphas of the three scales were 0.86, 0.81, and 0.79, respectively (data not presented).

One significant factor emerged from the work performance dimension, representing 61.2% of the total variance. This factor had four components, with factor loadings of the four items ranging from 0.59 to 0.80 (Table 4). Cronbach's alpha of the scale was 0.89 (data not presented).

## 4. Discussion

In this study job motivation contained three dimensions: institutional, societal, and individual work performance. This was in line with studies conducted in developing countries and Kenya where worker motivation was malleable and was affected by changes in the individual worker, work conditions, and broader social environment [12, 13].

In this study eight factors emerged from job motivation dimensions of health extension workers, namely, live conditions, access to infrastructure, community recognition, financial incentive, educational career, workload, better achievement in work, and supervisor encouragement.

Studies revealed that access to appropriate infrastructures and living conditions can significantly improve health workers morale [5].

In this study community recognition was found to be one of health extension workers job motivation scales. Studies conducted in Vietnam, Kenya, and developing countries showed that the value which the community gives to community health workers was found as category of job motivation scale [9, 12, 13]. The possible explanation could be high participation of the community in the identification of their common health problems and implementation of health-sector activities leads to greater acceptance and use of services, which may also make health extension workers think as they are getting better recognition from the community and develop sense of self-altruism. Experience elsewhere shows that the greater the participation of the community, the greater the acceptance and use of services and the lesser the demand for expensive curative services [14].

In the current study financial incentive was found to be one emerging factor of health extension workers job motivation scale. It is clear that financial incentives are highly influential in health worker motivation; furthermore financial incentives are factors that can significantly improve morale [10].

Again educational career was found as category for health extension workers job motivation scale. Study conducted in Vietnam showed that training was a motivating factor for workers [9]. The possible explanation could be that training enables health extension workers to take on more demanding duties and to achieve professional advancement as well as allow them to cope better with the requirements of their job. This indicates that refresher courses or trainings to reinforce current knowledge of the health extension workers are mandatory.

This study also showed that workload and better work achievement are the other categories of health extension workers job motivation scale. The study conducted in developing countries showed that the amount of work was found to be one category for job motivation scale [13]. Health planners or managers should understand how much workload affects the coverage, quality, and equity of health services provided by HEWs to their communities as heavy/overburden workloads have a tendency to discourage HEWs in quality equitable service provision.

## 5. Conclusion

This job motivation scale can be used or adapted to measure job motivation of community health workers and other professionals who are working in different settings. Further implementation research is needed in differing setting to further refine the scale.

## Figures and Tables

**Table 1:** The sociodemographic characteristics of health extension workers in Gamo-Gofa Zone, Southern Ethiopia, 2013.

Variables	Frequency (*n* = 301)	Percent (%)
Marital status		
Married	196	65.1
Not married	105	34.9
Religion		
Orthodox Christian	106	35.2
Protestant Christian	185	61.5
Muslim	10	3.3
HEWs in the current health post		
One	32	10.6
Two	237	78.7
Three	18	5.9
Four	14	4.8
Additional source of income^*^		
Yes	210	69.8
No	91	30.2
Current educational level		
Attended 10th grade	216	71.8
Above 10th grade	85	28.2
Service year	Average 5.1 (SD ± 2.1) years	
Age	Average 26.2 (SD ± 3.3) years	

^*^Additional source of income = source from farming, trade, or constant support from relatives.

**Table 2:** Factor loadings of the items used to measure the institutional support dimensions of job motivation by health extension workers, Southern Ethiopia, 2013.

Items	Education career	Workload	Financial incentive	Supervisor encourage
Opportunity for upgrade and trainings	0.72			
Opportunity of refresher trainings	0.69			
No skill gap	0.62			
Ability to deal with outbreaks	0.65			

No shortage of materials and equipment		0.72		
Duties assigned are not too much		0.76		

Satisfaction on monthly salary			0.83	
Satisfied on field allowance			0.68	
Satisfied on hardship allowance			0.65	
Satisfied on housing benefits			0.71	

Supervisor encourages in decision				0.66
Supervisor encourages to work hard				0.68
Supervisor gives supportive feedback				0.61
Supervisor makes HEWs feel valued				0.62

**Table 3:** Factor loadings of the items used to measure the societal support dimensions of job motivation by health extension workers, Southern Ethiopia, 2013.

Items	Community recognition	Infrastructures access	Living condition
Good relationship with local community	0.67		
The community has good perception for the HP	0.77		
All people in the village use the HEP services	0.66		

Accessible schools for children		0.76	
Convenient roads to move in the community		0.70	
No transportation problem		0.69	
Families are living in nearest place		0.74	
Water sources are in nearest place		0.72	

Living within 1 km far from HP			0.75
Living within 1 km far from water source			0.70
Living within 1 km far from market place			0.73
Living in compound of HP			0.72

**Table 4:** Factor loadings of the items used to measure the work performance dimension of job motivation by health extension workers, Southern Ethiopia, 2013.

Items	Better achievement
Achieve more in my work	0.80
Satisfied with the work done	0.71
Working hard for provision of quality service	0.64
Frequent visit to provide community services	0.59
